# Optical strategies for in vivo retinal ganglion cell imaging

**DOI:** 10.1007/s44258-025-00066-2

**Published:** 2025-11-17

**Authors:** Justin Chen, Raymond Fang, Xiaorong Liu, Hao F. Zhang

**Affiliations:** 1 Department of Biomedical Engineering, Northwestern University, Evanston, IL USA; 2 Department of Biology, University of Virginia, Charlottesville, VA USA

**Keywords:** Retinal ganglion cell, Scanning laser ophthalmoscopy, Optical coherence tomography, Two-photon imaging, Calcium imaging

## Abstract

Retinal ganglion cells (RGCs) are essential in transmitting visual information from the retina to the brain, and their impairment has been linked to glaucoma and various neuro-ophthalmic diseases. In vivo imaging of RGC morphology and functionality is crucial for understanding the pathophysiology of retinal disease caused by RGC degeneration and their responses to treatments. This review provides a comprehensive overview of optical technologies suitable for in vivo RGC imaging. First, we compare scanning laser ophthalmoscopy, optical coherence tomography, and two-photon imaging and discuss their effectiveness in quantifying RGC damage in retinal disorders. Then, we discuss how functional vascular imaging techniques and specialized fluorophores, such as capQ and GCaMP, can be exploited to provide deeper insights into the physiology of RGCs. Lastly, we highlight the clinical translation of these imaging modalities, emphasizing handheld devices and clinical workflows to improve the image acquisition process. We also highlight the emerging role of machine learning, which automates tasks such as segmentation and disease classification to improve the efficiency of large data analysis.

**Graphical Abstract:**

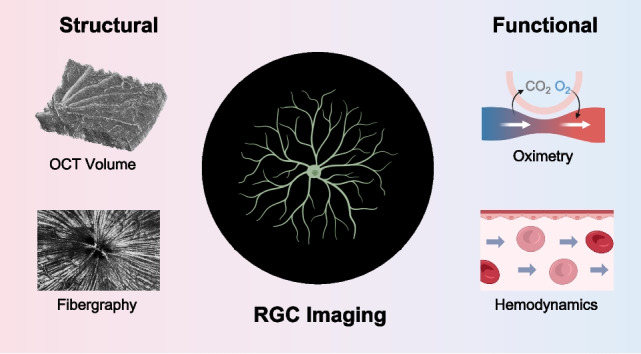

## Highlights

• Various optical modalities, such as SLO and OCT, have been developed to image RGCs and diagnose retinal neuropathies.

• Retinal oximetry and Doppler OCT reveal how oxygenation and blood flow affect the health and function of RGCs.

• Selective probes and calcium-sensitive dyes can be used to record cellular activity, death, and drug effects.

• Advances in device miniaturization and AI-driven analysis are accelerating the clinical adoption of in vivo RGC imaging.

## Introduction

**Fig. 1:**
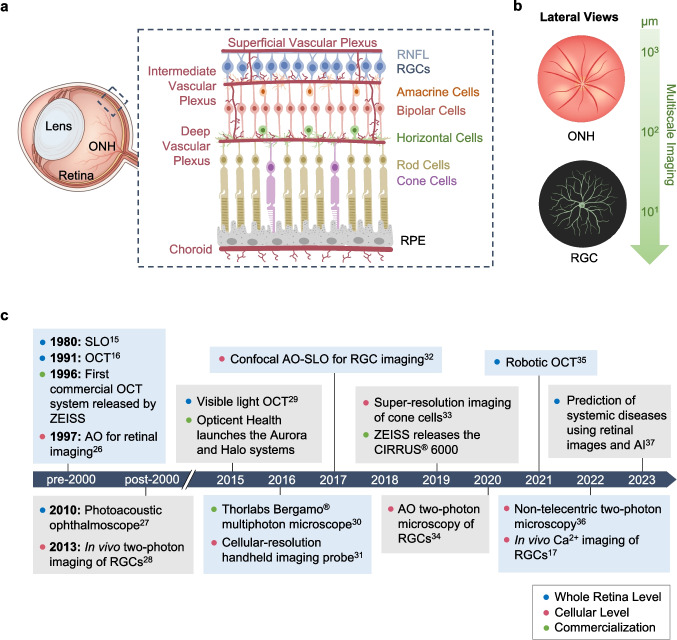
**a** Retinal circuitry illustrating the flow of visual signals through different neuronal layers of the retina. Vascular networks, known as plexuses, supply these cells with oxygen and nutrients to support their metabolic activities. Retinal ganglion cells are the final output neurons of this pathway, transmitting visual information to the rest of the central nervous system via the optic nerve head. ONH: optic nerve head, RGC: retinal ganglion cell, RNFL: retinal nerve fiber layer, RPE: retinal pigment epithelium. **b** Multiscale imaging enables the visualization of important retinal features, from the entire ONH to individual RGCs. **c** A timeline of key developments in the field of RGC imaging. Highlights include the invention of SLO and OCT, as well as the first use of two-photon imaging to visualize RGCs

More than 596 million people worldwide are at risk of vision loss owing to retinal diseases, such as glaucoma and diabetic retinopathy, many of which are characterized by retinal ganglion cell (RGC) degeneration and death [1]. RGCs primarily reside in the ganglion cell layer (GCL) and extend their axons to form the retinal nerve fiber layer (RNFL), which is crucial for relaying visual signals from the retina to the brain (Fig. 1a) [2, 3]. The function of RGCs is supported by blood vessels within the retinal plexuses [4]. In glaucoma, elevated intraocular pressure (IOP) often contributes to RGC loss and axonal degeneration [5, 6], which, in turn, leads to irreversible blindness [7]. In contrast, diabetic retinopathy results from retinal microvasculature dysfunction [8] secondary to elevated blood glucose levels, which creates an ischemic environment that impairs RGC function and survival [9].

As a result of their gradual onsets, these neurodegenerative retinal diseases often go unnoticed in their early stages. It is estimated that more than half of patients with conditions like glaucoma and diabetic retinopathy remain unaware of their conditions until significant vision loss occurs [10]. Therefore, early symptom detection provides opportunities to prevent or slow down the progression of subsequent neuropathy. Given its easy accessibility [11], the retina is an ideal target for non-invasive, high-resolution imaging [12–14]. Structural imaging modalities, such as scanning laser ophthalmoscopy (SLO) [15] and optical coherence tomography (OCT) [16], have been designed to visualize anatomical features within the retina at multiple scales, from the full optic nerve head to individual neurons (Fig. 1b). On the other hand, functional imaging techniques [17–19] aim to quantify the physiological properties of the retina through indirect methods, such as molecular labeling [20] and advanced signal processing [21, 22]. Examples include Doppler OCT [23], imaging-guided oximetry [24], and fluorescence imaging [25].

This review discusses key developments in optical retinal imaging (Fig. 1c) [15–17, 26–37], focusing on their working mechanisms and applications. We highlight several structural and functional optical imaging techniques, followed by an in-depth exploration of emerging techniques capable of achieving the resolution necessary for visualizing RGCs. Finally, we discuss ongoing challenges in refining existing optical systems, incorporating machine learning for improved diagnostics, and the potential for translational imaging.

## Structural imaging of RGCs

Numerous structural imaging modalities have been designed to visualize the diverse anatomical features of the retina, ranging from the optic disc to individual RGCs. While earlier forms of retinal imaging have encountered difficulties in resolving cellular-level structures [38], efforts have been focused on improving their spatial resolution, temporal resolution, and contrast [38, 39]. The emergence of cellular-resolution imaging modalities has allowed for quantifying the loss of neurons and detecting subtle morphological changes, such as dendritic pruning [40] and axonal thinning [41]. These biomarkers can serve as early indicators of neurodegeneration, enabling the prediction of central nervous system diseases, such as glaucoma, Parkinson's disease, and Alzheimer's disease [42, 43]. The rest of this section examines the leading structural retinal imaging modalities, focusing on their fundamental principles, recent advancements, and clinical relevance in detecting early signs of RGC degeneration. A summary of these modalities, along with their key features and applications, is provided in Table 1.

**Table 1:** Overview of different optical imaging modalities for retinal imaging

	Axial Resolution	Lateral Resolution	Scan Speed	Typical Wavelength	Advantages	Disadvantages	Refs.
Scanning Laser Ophthalmoscopy							
Confocal SLO	30 µm^†^—300 µm^*^	1.5 µm^†‡^—5 µm^*^	2 kHz^*^—16 kHz^†^	450 nm—850 nm	• High lateral resolution and good fluorescent contrast to visualize RGC structures	• Unable to provide depth-resolved information	[44–50]
Two-Photon SLO	10 µm^†^—130 µm^*^	1 µm^†‡^—20 µm^*^	8 kHz^*^—400 kHz^†^	700 nm −1300 nm	• Widely used for recording RGC and photoreceptor function • Good fluorescent contrast to visualize RGC structures	• Femtosecond laser source can be costly • Not many systems are commercially available • Unable to provide depth-resolved information	[51–57]
Optical Coherence Tomography							
SD-OCT	1.5 µm^†^—5 μm^*^	2^†^—15 μm^*^	100 kHz^*^—10 MHz^†^	550 nm—840 nm	• One of the highest axial resolutions for retinal imaging • Able to provide three-dimensional tomographic images to distinguish the retinal layers	• Experiences signal roll-off at greater imaging depths	[58–61]
SS-OCT	3 μm^†^—6 µm^*^	10 µm^†^—20 μm^*^	100 kHz^*^—40 MHz^†^	1050 nm—1350 nm	• Less signal roll-off compared to SD-OCT • Able to provide three-dimensional tomographic images to distinguish the retinal layers	• Tunable laser sources can be costly • Not many systems are commercially available	[58–60, 62–64]

^*^ Values are estimated from typical commercial or research systems. Reference systems include the following: Confocal SLO – refs. [45, 46], Two-Photon SLO – refs. [53, 56], SD-OCT – Zeiss Cirrus 6000, SS-OCT – Zeiss Plex Elite 9000

^†^ Achievable with advanced research systems. Values are provided to show current capabilities but may not necessarily be representative of standard imaging systems. Reference systems include the following: Confocal SLO – refs. [49, 50], Two-Photon SLO – refs. [51, 57], SD-OCT – ref. [61], SS-OCT – ref. [64]

^‡^ Resolutions are achievable with AO correction

### Scanning laser ophthalmoscopy

**Fig. 2:**
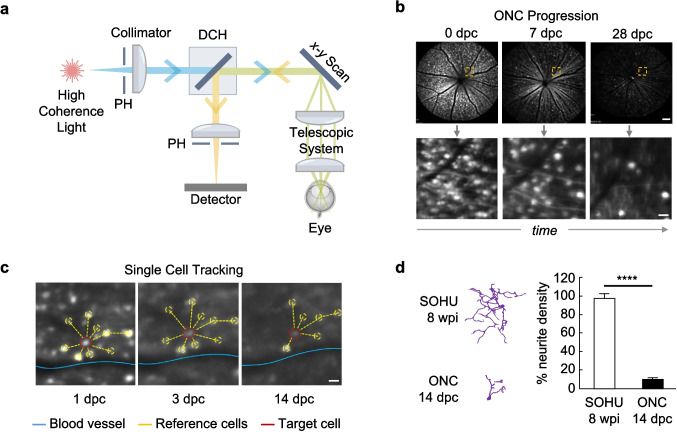
Scanning Laser Ophthalmoscopy. **a** Working principle of SLO. PH: pinhole, DCH: dichroic mirror. **b** Representative SLO images showing the degeneration of RGCs over one month in an ONC mouse model. ONC: optic nerve crush, dpc: days post crush. Scale bars: 100 μm (top), 20 μm (bottom). **c** SLO enables single cell tracking of RGCs in vivo to study migratory patterns following ONC. Blood vessels and neighboring cells can be used as landmarks for target cell identification. Scale bar: 20 µm. **d** Individual RGCs were segmented to compare their neurite densities following SOHU and ONC, with the ONC model showing a significant loss. SOHU: silicone oil induced ocular hypertension, wpi: weeks post injection. Panel **b** adapted with permission from ref. [70]. Panels **c** and **d** adapted with permission from ref. [17]

SLO is an in vivo retinal imaging technique first reported by R.H. Webb in 1980 [15]. It uses long-coherence length lasers that can be tuned across a broad spectral range, depending on the application needs [44, 65]. Wavelengths in the visible spectral range are better suited for imaging superficial retinal tissues, such as the RNFL, as they offer increased spatial resolution [66]. Longer near-infrared (NIR) light can penetrate biological tissues more effectively [67], rendering it advantageous for the visualization of deeper layers, such as the choroidal circulation [65]. In a typical SLO system (Fig. 2a), a beam splitter directs the incident light to a two-dimensional scanner that translates it across the retina in a raster pattern [68] to achieve the desired field-of-view (FOV). A telescopic system comprised of two separate lenses may also be placed before the eye to control the magnification of the target tissue [69]. In confocal SLO, the reflected light from the retina travels back to the system, passing a pinhole aperture before reaching the detector. The pinhole prevents the passage of stray and off-focus light to the detector. By only recording in-focus reflection, the contrast and resolution of the final image are enhanced, allowing for clearer visualization of the intended structures.

One common application of SLO is to image the optic disc [71], a vascular region of the retina where the RGC axons exit the eye. This region contains the entry point of the central retinal artery and the exit point of the central retinal vein, which supply and drain the inner retina of blood to support metabolic demands [72]. In a longitudinal study investigating the mouse model of optic nerve crush (ONC) [70], cellular-resolution SLO was used to track the apoptosis of RGCs for one month. ONC is an experimental procedure in which the optic nerve is mechanically compressed to induce localized axonal injury [73–75]. This model produces rapid and reproducible RGC degeneration, making it a widely used tool for studying mechanisms of optic neuropathy and evaluating potential neuroprotective therapies. Representative images of the optic disc throughout this process are shown in Fig. 2b [70]. The corresponding decay curves of RGC intensities revealed a progressive decline in fluorescence over time, reflecting the gradual loss of labeled cells from the retina.

Building on its ability to visualize population-level changes, SLO also supports longitudinal tracking of individual RGCs, allowing for analysis of cell migration in disease models [17]. As illustrated in Fig. 2c, individual cells were tracked across multiple time points following ONC, using local blood vessels and surrounding reference cells as landmarks. To further compare disease progression between models, confocal imaging and segmentation were used to assess neurite morphology in the ONC and silicone oil-induced ocular hypertension (SOHU) model, which better mimics the progressive and chronic nature of glaucoma. In SOHU, silicone oil is injected into the anterior chamber, where it floats and blocks the pupil, thereby disrupting the flow of aqueous humor [76]. This leads to fluid buildup in the posterior chamber, sustained IOP elevation, and gradual RGC loss. As shown in Fig. 2d, confocal imaging revealed a dramatic reduction in neurite density in ONC eyes compared to the more gradual loss observed in SOHU retinas [17]. These findings highlight the utility of SLO for single-cell tracking in vivo and demonstrate how confocal microscopy can be used to validate these results by confirming structural differences.

### Optical coherence tomography

First introduced in 1991, OCT [16] has made a significant breakthrough in ophthalmology, serving as the “gold standard” in retinal imaging, with approximately 30 million procedures being performed annually [77–79]. Its main advantage over SLO is that it provides three-dimensional tomographic images [80], which allows for a more comprehensive reconstruction encompassing a broader range of depths. This expanded coverage is crucial for understanding the interactions between RGCs and other neurons in the visual pathway, such as bipolar cells, which provide excitatory input, and amacrine cells, which modulate their activity through inhibitory signals [81]. Moreover, the ability to provide tomographic images allows for the quantification of RNFL thinning in glaucoma [82], detection of macular edema in diabetic retinopathy [83], and identification of drusen or geographic atrophy in age-related macular degeneration [84].

**Fig. 3:**
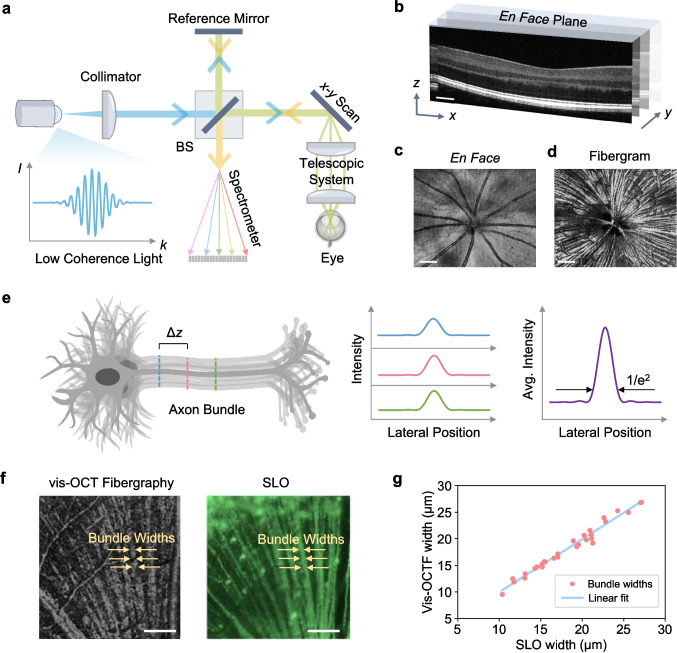
Optical Coherence Tomography. **a** Working principle of SD-OCT. BS: beam splitter. **b** Representative OCT B-scans of the human retina, distinguishing the different layers. Scale bar: 300 µm. **c** An example of an *en face* image, taken from a mouse retina. Scale bar: 100 μm. **d** The corresponding fibergram of the *en face* image shows individual retinal nerve fibers. Scale bar: 100 μm. **e** Workflow for quantifying axon bundle widths using OCT fibergraphy. Intensity profiles are extracted at evenly spaced axial positions along the bundle and averaged. The width is defined at the point where the averaged intensity falls to 1/*e*^2^ of the peak. **f** Close-up images of nerve fibers taken using vis-OCT fibergraphy and SLO. A set of widths from one fiber bundle is labeled. Scale bars: 100 µm. **g** Bundle widths from 30 RGC fibers were measured using both vis-OCT fibergraphy and SLO, showing strong agreement based on a linear regression analysis. Panels **c** and **d** adapted with permission from ref. [87]. Panels **f** and **g** adapted with permission from ref. [88]

The working principle of OCT (Fig. 3a) is similar to ultrasound imaging. In its simplest design, low-coherence light is transmitted through a Michelson interferometer, which splits the light into two paths: a sample arm and a reference arm [85]. In the sample arm, light is illuminated through the pupil of the eye, generating a set of reflected beams that travel back from different retinal layers. Similarly, light reflects off a mirror in the reference arm. When the light from the sample and reference arms is recombined, a spectral interference pattern is generated in modern spectral-domain (SD)-OCT and swept-source (SS)-OCT. The *k*-space distribution of the interference pattern enables depth-resolved imaging [86].

While the Michelson interferometer is commonly used in OCT, some systems employ a Mach–Zehnder interferometer, which can also support balanced detection schemes to improve signal quality and suppress noise [89]. Balanced detection relies on the interference signals being π out of phase at two outputs, enabling common-mode noise to be effectively canceled when the signals are subtracted [90]. Michelson systems achieve this using the two output ports of a single beam splitter, while Mach–Zehnder interferometers use two beam splitters to generate spatially separated, complementary signals. This separation enables greater flexibility in managing optical path lengths and facilitates the use of dual spectrometers. In a recent study by Rubinoff et al. (2022), balanced detection was implemented in a visible light optical coherence tomography (vis)-OCT system based on a Mach–Zehnder interferometer and used the spectrally encoded relative intensity noise to calibrate the spectrometers with subpixel accuracy [91]. This approach enabled precise phase alignment between outputs, significantly reducing the noise floor by up to 20.5 dB and achieving a correlation coefficient of 0.99 between signals. The tradeoff, however, is that Mach–Zehnder interferometers require more careful alignment and calibration compared to the simpler Michelson layout.

The Fourier transform of the interference pattern encodes the intensity of light reflected from a specific location in the retina, with structural information for that location encoded as an A-line. For point scanning OCT, the incident OCT beam is scanned with a galvanometer to image a region of interest. A set of A-lines is used to reconstruct cross-sectional images, known as B-scans [92]. A three-dimensional tomographic image can be reconstructed by merging multiple B-scans in the *y*-direction (Fig. 3b). The *x–y* plane, known as an *en-face* plane, provides a top-down view of the retina, similar to that of traditional fundus photography or SLO image.

SD and SS systems are capable of scanning at a rate of several MHz [93]. SD-OCT usually employs a broadband NIR superluminescent diode for illumination, typically operating with a center wavelength near 840 nm, coupled with a spectrometer for detection [58]. The recent development of vis-OCT has shown that light in the visible spectral range can also be integrated into SD-OCT to acquire higher axial resolution images [94]. In contrast, SS-OCT employs a tunable laser that rapidly sweeps through a range of wavelengths in succession and is paired with a single-photon detector [62]. For OCT design, longer wavelengths enable deeper tissue penetration and reduced scattering, which is well suited for visualizing deeper layers of the retina. In contrast, shorter wavelengths result in higher axial resolution with increased scattering, often leading to better contrast between the superficial layers of the retina.

One prominent application of OCT is the visualization and analysis of RGC axon bundles, known as OCT fibergraphy [95]. Rather than measuring the bulk thickness of the retinal nerve fiber layer, which is currently the standard method for tracking the progression of glaucoma [96], OCT fibergraphy seeks to quantify the morphological parameters of individual axon bundles, such as their width and cross-sectional area [97]. Examples of a vis-OCT-acquired *en-face* image of the mouse retina and its corresponding fibergram are shown in Fig. 3c and d, respectively [87]. To assess the accuracy of vis-OCT fibergraphy, a separate study [88] compared axon bundle widths measured from vis-OCT fibergraphy and fluorescence SLO images acquired in the same retinal region of transgenic mice expressing yellow fluorescence protein in RGCs. As shown in Fig. 3e, bundle widths were quantified by sampling lateral intensity profiles at multiple axial positions along each bundle, averaging them, and determining the width at which the signal is 1/*e*^2^ of the peak. Magnified images from both imaging modalities are shown in Fig. 3f, with individual bundle widths labeled for direct comparison. Quantitative analysis across 30 bundles revealed a strong linear correlation (R^2^ = 0.977) between the two techniques (Fig. 3g), supporting the reliability of vis-OCT fibergraphy for in vivo measurement of single RGC axon bundle morphology.

Various mathematical models, including fractal analysis and Sholl analysis, have also been deployed to analyze the spatial patterns and connectivity of axons from OCT fibergraphy [98, 99]. Fractal analysis measures the complexity of RGC axon bundle organization by calculating the fractal dimension, a parameter that describes how intricate a structure appears at different levels of magnification [99]. Sholl analysis, on the other hand, evaluates the spatial distribution of axon bundles by measuring the number of intersections with concentric circles centered at the optic nerve head, allowing for the assessment of how bundle density changes with distance [98]. Moreover, signal acquisition schemes such as temporal speckle averaging (TSA) can be used to enhance the clarity and resolution of images, providing higher contrast OCT fibergraphy [100, 101]. By averaging multiple OCT scans acquired over a short period, TSA reduces speckle noise and allows for a more accurate assessment of axonal integrity.

OCT fibergraphy has been widely used in animal models to detect early signs of retinal neurodegeneration. In mice, it has revealed axonal swelling and thinning of nerve fiber bundles following ONC, with reductions in width, height, and cross-sectional area correlating with progressive RGC soma loss [97, 102]. More recently, studies in tree shrews, whose vertically stratified nerve fibers and layered inner plexiform structure more closely resemble the human retina, have demonstrated the ability of OCT fibergraphy to resolve sublayer-specific changes, including disruptions in fiber stratification [100]. These findings support the use of OCT fibergraphy as a promising tool for detecting and monitoring conditions such as glaucoma in humans [103].

### Two-photon microscopy

**Fig. 4:**
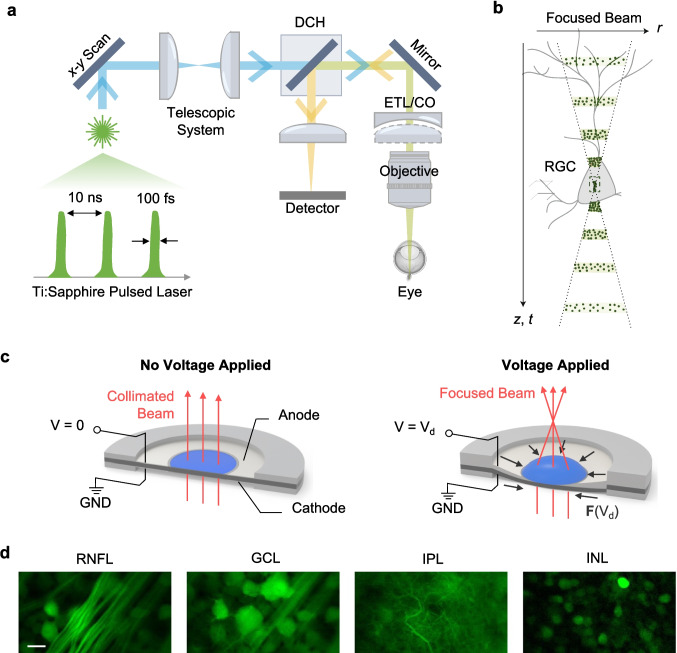
Two-Photon Imaging. **a** Working mechanism of a two-photon imaging system. ETL: electronically tunable lens, CO: concave offset lens, DCH: dichroic mirror. **b** The intensity distribution around the focal point of a two-photon system, in which the probability of simultaneous absorption events is highest at the center. $r$: lateral coordinate, $z$: axial coordinate, $t$: time. **c** Working mechanism of an ETL. An applied voltage ${V}_{d}$ induces a compressive force ${\varvec{F}}({V}_{d})$, which deforms the lens membrane and alters the curvature of the fluid interface. This change in curvature modifies the power of the lens. GND: ground. **d** Various cross sections, each depicting a different retinal layer, are able to be visualized by adjusting the ETL placed adjacent to the objective lens. RNFL: retinal nerve fiber layer, GCL: ganglion cell layer, IPL: inner plexiform layer, INL: inner nuclear layer. Scale bar: 20 µm. Panel **b** adapted with permission from ref. [111]. Panel **c** adapted with permission from ref. [112]. Panel **d** adapted with permission from ref. [34]

In recent years, two-photon microscopy has gained traction in RGC imaging, offering advantages such as enhanced contrast and high signal-to-noise ratio [104] compared to SLO. It relies on the nonlinear excitation of fluorophores within the tissue, achieved by the simultaneous absorption of two lower-energy photons in the NIR spectral range [105, 106]. In addition, illuminating with longer wavelengths relative to the visible spectra reduces the optical scattering in biological tissue [107–109]. A typical setup, shown in Fig. 4a, consists of a Ti:Sapphire laser source that emits ultrashort pulses in the femtosecond range, as well as a dichroic mirror to separate the excitation and fluorescence emission paths [51, 110].

One of the most apparent differences between the optical setups of two-photon and confocal microscopy is that the former does not require a pinhole aperture [113]. In confocal microscopy, a pinhole is crucial for rejecting out-of-focus light. This mechanism improves image contrast by selectively allowing the light emitted from the focal plane to reach the detector. However, in two-photon microscopy, fluorescence emission occurs in a specific femtosecond time frame in which the absorption occurs [114]. Due to this constraint, fluorescence excitation only occurs within a specific focal volume (Fig. 4b), effectively accomplishing the same task as a pinhole [111]. This intrinsic depth confinement reduces background noise and enhances contrast without sacrificing signal intensity.

Two-photon microscopy is often combined with SLO to enhance the imaging of RGCs in vivo. SLO offers the advantage of an easily adjustable FOV, typically around 30 degrees, which corresponds to 2 mm on the mouse retina [115]. Two-photon microscopy complements this by providing optical sectioning with depths up to several hundred micrometers [109], sufficient to capture the inner retinal layers and visualize fine cellular structures, such as nerve fibers, microglia, and RGC somas [34]. These hybrid systems minimize photodamage through their use of NIR wavelengths, which reduce phototoxic effects due to their lower energy levels. Moreover, the nonlinear excitation process confines excitation to the focal plane, avoiding unnecessary exposure of surrounding tissues [52].

In addition to combining SLO and two-photon microscopy, many current two-photon retinal imaging systems also incorporate an electronically tunable lens (ETL) paired with a concave offset (CO) lens to enable axial scanning. The ETL modulates focal length by applying a voltage-induced compressive force ${\varvec{F}}({V}_{d})$ to a fluidic lens, deforming its elastic membrane and altering the curvature of the refractive interface (Fig. 4c) [112]. Moreover, the CO lens introduces a fixed divergence to the incoming beam, effectively extending the axial tuning range of the ETL and improving depth coverage. This configuration allows rapid refocusing across different retinal layers, from the RNFL and GCL to deeper structures such as the inner nuclear layer (INL), without physically moving the objective lens (Fig. 4d) [34]. In a study by Bar-Noam et al. (2016), the axial shift achieved by tuning the ETL was quantified through three approaches: a simplified paraxial model, a detailed ray-tracing Zemax model, and direct experimental measurements using fluorescent beads [116]. Notably, the axial shifts inside the eye were found to be approximately 4 times smaller than those measured without the eye due to the focusing effect of the mouse crystalline lens.

**Fig. 5:**
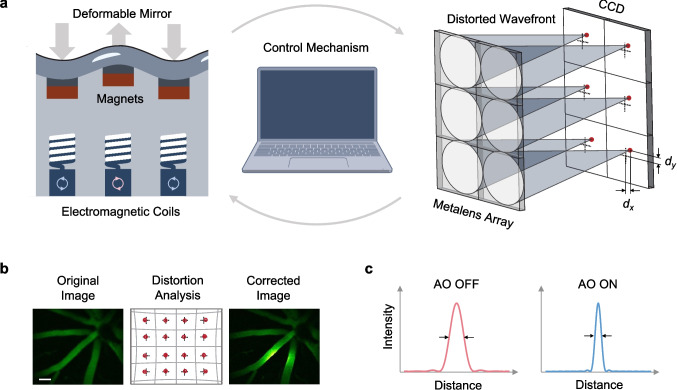
Principle of adaptive optics. **a** An illustration of an AO system consisting of a deformable mirror (left) and a Shack-Hartmann sensor (right). **b** An image of the optic disc acquired by two-photon fluorescence microscopy before (left) and after (right) wavefront correction. Scale bar: 100 µm. **c** Point spread functions before and after AO correction. Panel **b** adapted with permission from ref. [51]

Lastly, it is important to note that adaptive optics (AO) is typically used in two-photon retinal imaging systems to correct for optical aberrations caused by the cornea, lens, and other ocular components. With AO, two-photon retinal imaging systems have been able to resolve cellular structures, such as somas and axons, as little as a few micrometers apart [34, 51, 117, 118]. Figure 5a shows the working mechanism of a typical aberration sensor, known as a Shack-Hartmann sensor [119]. When a distorted wavefront passes through this sensor, a set of lenslets focuses different portions of the original beam onto a charge-coupled array device, creating an array of spot patterns. By analyzing the displacement of these spots from their ideal positions, the sensor can quantify the location of the original aberrations. This information is then relayed to a deformable mirror [120], which dynamically adjusts its components to counteract the aberrations by adjusting the optical path length [121]. Together, the wavefront sensor and deformable mirror form a closed-loop system that is mediated by a computational algorithm.

The effects of AO can be observed by comparing images of the optic disc acquired by two-photon fluorescence microscopy [51] (Fig. 5b). In the original image, the vasculature exhibits reduced contrast, with edges appearing less sharp and finer structural details obscured due to aberrations caused by both the optical system and the eye. However, these aberrations are corrected with AO, resulting in a significantly improved definition of vasculature structures. The enhancement in spatial resolution is quantitatively demonstrated by a reduction in the full width at half maximum of the point spread function (Fig. 5c). This parameter describes the ability of AO-integrated systems to discriminate two closely spaced objects, facilitating precise measurements of structures close to the diffraction limit [122].

## Functional imaging of RGCs

The primary goal of functional imaging is to expand the clinical applications of structural imaging by measuring dynamic physiological processes, such as blood flow, metabolic activity, and neural response [123]. This approach is particularly valuable for evaluating the metabolic consumption of RGCs [124], enabling early detection and intervention for conditions such as glaucoma, where functional impairments often precede visible structural damage. In this section, we delve into the diverse applications of functional retinal imaging, focusing on fundamental principles, methodologies for data analysis, and the potential implications for personalized treatment.

### Hemodynamics and retinal oxygen metabolism

**Fig. 6:**
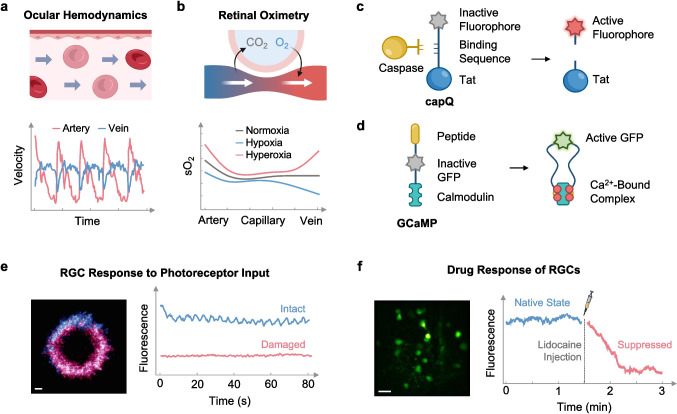
In vivo functional imaging of retinal vasculature and RGCs. **a** Hemodynamic parameters, such as axial flow velocity, in retinal blood vessels can be quantified noninvasively using Doppler OCT. **b** Acquisition of sO_2_ data by fitting the absorption spectrum from vis-OCT to measured spectrums of oxygenated and deoxygenated hemoglobin. This data can be used to quantify the changes in oxygen saturation as blood flows from artery to vein within the retina. **c** The capQ complex specifically binds to caspases released by dead RGCs, activating its NIR fluorophore. **d** GCaMP is another fluorescent complex that activates in the presence of calcium. **e** One application of calcium imaging is assessing the activity of RGCs in response to photoreceptor input. Scale bar: 150 µm. **f** Calcium imaging can also evaluate cellular drug response induced by lidocaine. Scale bar: 50 µm. Panel **e** adapted with permission from ref. [132]. Panel **f** adapted with permission from ref. [51]

Through advanced signal processing techniques, OCT can also measure physiological parameters such as blood flow and oxygen saturation [125, 126]. Many specialized forms of OCT have been developed to better understand the eye's metabolic demands and perfusion characteristics. For instance, OCT angiography was designed to enhance the contrast of vascular regions by distinguishing blood vessels from static tissues [127]. It relies on the motion contrast generated by moving blood cells and operates without exogenous contrast agents. Doppler OCT, on the other hand, can provide quantitative information about ocular hemodynamics (Fig. 6a) [23, 128] and the elastic properties [129] of various retinal layers. It measures the Doppler shift experienced by the signal, where motion along the direction of the incident light causes a detectable phase shift in the backscattered signal, enabling sensitive measurements of blood flow velocity in the direction of the incident light [130]. The relationship between blood velocity and parameters acquired from Doppler OCT can be summarized by Eq. (1), where $\Delta \phi$ represents the phase shift between sequential A-scans, $T$ is the time between A-scans, ${\lambda }_{0}$ represents the center wavelength, $n$ is the refractive index, and $\alpha$ is the angle formed between the directions of illumination and flow [131]. However, $\alpha$ is often difficult to measure accurately in vivo due to the complex and variable orientation of blood vessels relative to the incident beam.

1 $$v=\frac{\Delta\phi\lambda_0}{4\pi nT\cos\alpha}$$

Many recent studies have leveraged the advantages of visible light to acquire functional data, enabling more accurate assessment of physiological parameters [66, 133, 134]. By utilizing shorter wavelengths, vis-OCT achieves improved resolution, resulting in higher contrast for blood flow. Additionally, the differential absorption of visible light by oxygenated and deoxygenated hemoglobin allows for measuring oxygen saturation (sO_2_) for individual vessels [135]. One study [136] exemplified this by employing vis-OCT to quantify blood flow and sO_2_ levels in rats subjected to systemic hypoxia. In this study, the flow rate was calculated by multiplying the velocity ($v$) by the total cross-sectional area ($A$) of either the arteries or veins. Furthermore, the sO_2_ levels were calculated by measuring the attenuation coefficient as a function of wavelength, with the reconstructed OCT signal fit to Eq. (2).

2 $$I^2\left(\lambda\right)=I_0^2\left(\lambda\right)R_0r\left(\lambda\right)\exp\left[-2d\mu_{HbO_2}\left(\lambda\right)\times sO_2-2d\mu_{Hb}\left(\lambda\right)\times\left(1-sO_2\right)\right]$$

Here, $I\left(\lambda \right)$, the recorded intensity at the posterior vessel wall, is an oxygen- and wavelength-dependent variable measured in response to a broadband light source [136, 137]. Additionally, ${I}_{0}(\lambda )$ represents the incident intensity, ${R}_{0}$ is the reflectance of the reference arm, $r(\lambda )$ denotes the reflectance of the vessel wall, $d$ is the diameter of the vessel, and ${\mu }_{i}\left(\lambda \right)$ is the optical attenuation coefficients of oxygenated (HbO_2_) and deoxygenated (Hb) hemoglobin.

To analyze the implications of different retinal oxygenation states, a subsequent investigation [138] employed these methods to record the sO_2_ levels of rats undergoing alternating periods of normoxia, hypoxia, and hyperoxia (Fig. 6b). Corresponding data revealed significant changes in oxygenation from artery to vein, with veins showing the greatest variation, ranging from 50% in hypoxia to 85% in hyperoxia. In contrast, capillary sO₂ changed by less than 10%, indicating the tight regulation of oxygen exchange at the capillary level. Since capillaries are most abundant in the inner retina, where RGCs reside, this stability ensures a consistent oxygen supply to meet the high metabolic demands of these cells [139]. Meanwhile, larger venous fluctuations may reflect the cumulative oxygen exchange across the retina [140]. This dynamic regulation can help identify hypoxic stress or metabolic dysfunction underlying RGC damage in diseases like glaucoma and diabetic retinopathy and may serve as biomarkers for evaluating protective therapies.

### Neural activity

Fluorescence imaging is a powerful tool for investigating neural activity in the retina, providing insights into processes such as apoptosis, oxidative stress, and energy metabolism [25, 141]. One major application is detecting RGC apoptosis, a critical marker of neural dysfunction and degeneration [142]. For in vivo applications, apoptosis-specific probes have been engineered to selectively label dying RGCs without interfering with the activity of healthy cells [143, 144]. One such probe, capQ [144], integrates a cell-penetrating Tat peptide, a protease recognition sequence, and an NIR fluorophore (Fig. 6c). While capQ remains optically inert in active cells, its fluorophore is activated upon cleavage by proteases such as caspases 3 and 7, which are only secreted by RGCs undergoing programmed cell death [145]. This selective activation enables real-time visualization of neural degeneration [146]. Other tools include annexin V-based fluorescent markers, which bind to phosphatidylserine residues externalized during early apoptosis [147], and mitochondrial membrane potential-sensitive dyes, such as JC-1 or tetramethylrhodamine ethyl ester, which can detect early stages of apoptosis by identifying disruptions in mitochondrial function [148]. These diverse probes have been validated in animal models of glaucoma against well-established methods for identifying apoptotic RGCs, such as the TUNEL assay, which conjugates fluorescently labeled nucleotides to the 3’ ends of DNA fragments secreted by dead cells [146, 149, 150]. The safety and efficacy of these probes have been evaluated by over 30 clinical trials [144] with applications across a variety of systemic conditions. One such trial, focusing on the intravenous delivery of annexin V to detect apoptotic retinal cells, has advanced to phase II testing, ultimately highlighting its promise for clinical translation [151].

Another widespread fluorescence imaging technique is calcium imaging, which records the responses of calcium (Ca^2+^) sensitive fluorescent indicators as proxies for cellular activity, such as neuronal firing or synaptic transmission [152]. An example of such an indicator is GCaMP, a chimeric assembly consisting of three subdomains: green fluorescent protein (GFP), calmodulin, and a peptide chain derived from myosin light chain kinase (Fig. 6d) [153]. In response to light stimulation of photoreceptors, synaptic input elevates intracellular Ca^2+^ in RGCs, which causes binding interactions at the calmodulin domain [154]. This binding induces a conformational change, resulting in the GFP emission of 510 nm light. In a recent study [17], in vivo GCaMP-mediated calcium imaging was used to compare the ON/OFF activities of RGCs between ONC and glaucomatous models of mice. The ON state refers to RGCs that increase their firing rate in response to light onset, while the OFF state is activated when light intensity decreases [155]. Despite elevated intraocular pressures and optic nerve injuries in both the ONC and glaucoma models, significant differences in pathological mechanisms were observed [17]. For instance, it was found that RGCs predominantly converted to the OFF state after ONC, while most ON-RGCs retained their identities in the glaucomatous model. Moreover, GCaMP signals showed a rapid decline of viable RGCs in the ONC model. In contrast, cells in the glaucoma model experienced a slower degenerative process, with a significant population remaining after eight weeks. Overall, this study demonstrated how GCaMP-based calcium imaging can elucidate distinct RGC functional states, revealing the shortcomings of ONC as a model for glaucoma pathology.

Calcium imaging also serves as an effective method for assessing RGC response to external patterned stimuli [156]. In one study [132], ChrimsonR, a red-shifted channelrhodopsin, was used to investigate the activation of RGCs in the fovea of a living primate. ChrimsonR functions as an optogenetic actuator [157], enabling the control of neuronal activity in response to light stimulation. By co-expressing ChrimsonR with the calcium indicator GCaMP6s, researchers used AO-SLO to monitor calcium influx during patterned light stimulation [132]. When exposed to a 0.2 Hz patterned grating stimulus at a power of 12.5 mW/cm^2^, ChrimsonR-expressing RGCs exhibited a significant influx of Ca^2+^. The foveal ring was divided into two regions to validate the optogenetic activation. Photoreceptor input was eliminated in the upper region using a high-intensity 730 nm femtosecond laser delivering 55 fs pulses with an average power of 4.48 W/cm^2^, while the lower region was left intact. Subsequent flickering light stimulation, delivered at intensities sufficient to activate photoreceptors (mean Luminance 0.75 mW/cm^2^) but below the optogenetic threshold, produced periodic responses only in the intact region. However, at higher intensities targeting ChrimsonR, optogenetic Ca^2+^ influx was detected in the damaged photoreceptor region, confirming direct RGC activation (Fig. 6e).

Lastly, calcium imaging can be used to study the pharmacodynamics of neuroactive drugs, such as sodium and potassium channel blockers, focusing on their mechanisms of action, dose–response relationships, and time course of effects. A recent study [51] developed a two-photon fluorescence microscope with AO to observe alterations in RGC activity in mice following the retro-orbital administration of 10 µL of 2% lidocaine into the non-imaged eye. Lidocaine is a local anesthetic that blocks sodium channels to inhibit nerve conduction and prevent pain signaling [158]. It is commonly used in ophthalmology to numb the eye for invasive procedures, including glaucoma and cataract surgeries [159]. In this study, it was found that injection of lidocaine causes a rapid decrease in the relative fluorescence of RGCs, indicating sodium channel blockade and inhibition of action potential generation [160]. Fluorescence intensity returned to baseline levels within a minute, confirming that the dose of lidocaine effectively suppressed neural activity (Fig. 6f). These findings complemented those of a previous investigation [161], which compared the pharmacological effects of tetrodotoxin (TTX), another sodium channel blocker, on ON and OFF RGCs. While both RGCs express high-voltage-activated (HVA) calcium channels, only OFF RGCs express low-voltage-activated (LVA) calcium channels. Thus, OFF RGCs are also activated by hyperpolarization, leading to rebound excitation when the membrane potential returns to resting levels. Simultaneous two-photon calcium imaging and patch-clamp recordings revealed that the administration of 0.1 µM TTX not only blocks action potentials in both ON and OFF RGCs, but also diminishes the rebound-induced calcium influx in OFF RGCs, suggesting that TTX affects both HVA and LVA calcium channels.

## System integration and clinical translation

Optimizing retinal imaging devices and advancing computational techniques have accelerated the clinical adoption of in vivo RGC imaging. Handheld and compact imaging systems [162, 163] have addressed longstanding challenges related to motion artifacts [164] and patient accessibility. At the same time, novel optical methods, such as two-photon microscopy [165], have expanded diagnostic and therapeutic capabilities. Simultaneously, machine learning has served as a valuable tool for automated retinal structure and disease analysis, with algorithms designed to segment RGC images and classify pathologies [166, 167]. This section examines these developments and their implications for integrating RGC imaging into routine clinical practice.

### Device optimization

**Fig. 7:**
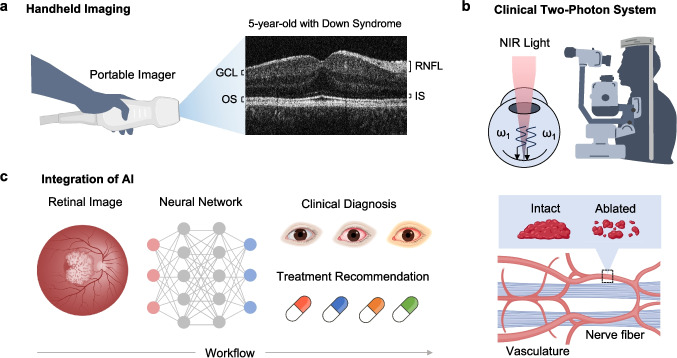
Systemic integration and clinical translation. **a** Handheld platforms may facilitate RGC imaging in specialized patient populations. An example image, taken from a 5-year-old child with Down syndrome, is shown to the right with the RGC and photoreceptor layers labeled to the side. RNFL: retinal nerve fiber layer, GCL: ganglion cell layer, IS: inner photoreceptor segment, OS: outer photoreceptor segment. **b** While in vivo two-photon retinal imaging is a relatively new field, its safe implementation in the clinic is currently being investigated by several early-stage trials. One application is image-guided microsurgery, where two-photon laser pulses can be used to ablate blood clots. **c** Incorporating artificial intelligence may prove useful in interpreting retinal images, ultimately leading to more accurate clinical diagnoses and treatment recommendations. Panel **a** adapted with permission from ref. [175]

Compact [45] and handheld [168] imaging platforms have addressed the fixation challenges of traditional systems, making RGC and photoreceptor imaging more accessible for infants, young children, and individuals with motor impairments [169, 170]. One strategy for enhancing portability is to replace the traditional galvanometer-based scanner with a micro-electromechanical (MEMS) scanner, a compact device that uses micromirrors to steer optical beams through electrostatic, electromagnetic, or piezoelectric actuation [171, 172]. Clinical trials in pediatric populations and studies in animal models [170, 173] have demonstrated that handheld OCT platforms can reduce motion artifacts while achieving axial resolutions as fine as 4.5 µm [174], enabling precise measurements of different retinal layer thicknesses from the ganglion cell layer to the retinal pigment epithelium, even in patients with involuntary eye movements [175]. Figure 7a shows a representative OCT image of a handheld device from a 5-year-old patient with down syndrome, with each of the main retinal layers clearly resolved. Using handheld OCT, previous studies were also able to quantify RGC density in wildtype horses [176] and photoreceptor density in children as young as 14 months of age [31].

Another direction in device optimization is the integration of two-photon microscopy into existing clinical SLO, OCT, and fundus imaging systems to facilitate diagnostic and therapeutic interventions. Though current two-photon studies have largely been limited to animal models, multiple early-stage clinical trials are already underway [165, 177], with applications ranging from transplantation of stem cell-derived RGCs [178] to quantification of RGC apoptosis [179] and laser-guided microsurgery [180]. One study, for instance, showed that femtosecond laser ablation can trigger the production of novel fluorescent molecules within biological tissues, effectively serving as in vivo labeling agents [181]. These compounds exhibit distinct spectral and temporal fluorescence properties, characterized by emission peaks beyond 500 nm and Lifetimes of approximately 1ns, three times shorter than those of standard fluorescent proteins, such as EGFP and tdTomato [20]. In a subsequent study [34], these properties were leveraged to delineate ablation boundaries in real-time during laser axotomy, a precise technique for severing axons, which has also been shown to stimulate retinal nerve regeneration, promote angiogenesis, and facilitate the dissipation of blood clots (Fig. 7b). Using a 740 nm femtosecond laser with 350 mW of power, an average ablation diameter of approximately 20 µm was achieved, a precision suitable for tracking degeneration in single RGC axons.

### Integration of machine learning

In recent years, machine learning has quickly gained momentum in ophthalmic imaging and modern medicine [182–184], guiding the detection, diagnosis, and treatment of retinal neuropathies (Fig. 7c) [185]. Numerous image classification algorithms have been designed and tested using publicly accessible SLO or OCT datasets to automate tasks, such as segmentation [166], cell counting [186], and classification [167]. To evaluate the performance of these models, a parameter known as the area under the receiver operating characteristic curve (AUROC) can be computed [183]. It plots the true positive rate against the false positive rate at various threshold settings, providing a single value between 0.0 and 1.0, where a higher value indicates better discrimination. An AUROC of 0.5 suggests no discriminative power, while a value closer to 1.0 indicates excellent classification performance. An example of machine learning in retinal imaging is demonstrated in a study introducing RETFound [37], a self-supervised foundation model pretrained on over 1.6 million retinal images and fine-tuned for various disease detection tasks, including glaucoma, diabetic retinopathy, and age-related macular degeneration. It incorporates specialized neural networks, such as masked autoencoders, to develop feature representations of retinal layers despite obscuring up to 75% of the image content during pretraining. Notably, RETFound achieved an AUROC of 0.943 on the Kaggle APTOS-2019 dataset, significantly higher than leading models, such as SSL-Retinal and SSL-ImageNet. This model also excelled in using retinal images to predict systemic diseases like myocardial infarction (AUROC 0.737) and ischemic stroke (AUROC 0.754). Another study [166] highlighted the development of RGC-Net, a deep learning model for automated reconstruction and quantification of RGCs. It is structured as a U-Net-based architecture with a ResNet-101 encoder and dual neurite and soma segmentation decoders, enhanced with skip connections to preserve spatial features and improve gradient flow. Trained on 166 RGC images, the model achieved dice similarity coefficients (DSC) of 0.691 and 0.850 for segmenting neurites and somas, respectively. The DSC measures the overlap between predicted and ground truth segmentation masks and is calculated as

3 $$\mathrm{DSC}=\frac{2\mathrm{TP}}{2\mathrm{TP}+\mathrm{FP}+\mathrm{FN}},$$

where TP, FP, and FN represent true positives, false positives, and false negatives, respectively [187]. RGC-Net outperformed traditional methods like U-Net and Panoptic FPN in foreground and background accuracy, particularly in handling complex neuronal morphologies such as branching and densely interconnected structures [166]. The automated pipeline significantly reduced analysis time from hours of manual tracing to just minutes while achieving a strong correlation (R^2^ = 0.904) with manual methods in measuring morphological properties such as soma length, width, and area.

## Outlook

In this review, we covered the principles and applications of several optical modalities for in vivo RGC imaging, including SLO [15], OCT [16], and two-photon microscopy [28]. We have also discussed how additional methods, such as Doppler OCT [23], retinal oximetry [134], and fluorescence imaging [154], can be used to quantify physiological parameters like blood flow, oxygen saturation, and cellular activity in the retina. These advancements provide novel information about early structural and functional alterations that precede significant neuronal loss in conditions such as glaucoma and diabetic retinopathy. Techniques like OCT fibergraphy [100] have enabled the detection of subtle changes in RGC axonal structure. At the same time, Doppler OCT and retinal oximetry have facilitated the quantification of ocular blood flow and oxygen saturation, offering biomarkers for early disease progression [128, 136]. The ability to noninvasively image RGCs in vivo has thus become indispensable in understanding the pathophysiology of these retinal neuropathies, allowing for early interventions that can slow or halt the degenerative processes before irreversible damage occurs, ultimately preserving vision.

Although significant advancements have been made in developing optical imaging techniques for in vivo RGC visualization, several challenges, including safety, accessibility, and reliability, remain to be addressed. Many of these imaging modalities, including two-photon microscopy [28, 177] and SS-OCT [62, 63], are still in their early stages of development, and additional long-term clinical studies will have to be conducted before they can be widely adopted into standard practice. Regulatory bodies, such as the American National Standards Institute (ANSI) and the Food and Drug Administration (FDA), are fundamental in defining safety thresholds and facilitating the approval process to bring these technologies into clinical settings [188]. For example, ANSI Z136.1–2000 outlines safety thresholds for ocular exposure to optical radiation, ensuring that imaging systems do not exceed permissible light levels that could harm retinal tissues [189]. Similarly, FDA guidelines emphasize rigorous preclinical testing, such as animal studies to assess phototoxicity thresholds and clinical trials to evaluate device performance, imaging accuracy, and potential adverse effects over extended use [190].

The ability to continuously monitor ocular health presents another ongoing challenge, especially for patients with chronic eye conditions who require routine care [191]. With the increasing role of AI and robotics in the automation of retinal imaging [192], specialized procedures can soon be brought to primary care settings, facilitating more frequent and accessible monitoring [35]. Implementing at-home screenings for ocular health may also be possible with the development of cost-effective equipment and telemedicine [193]. Although this remains a relatively new concept, a few at-home OCT prototypes have begun clinical testing [194, 195], with one company having already achieved a breakthrough device designation from the FDA [196]. This designation expedites the regulatory review process, allowing for faster 510(k) approval and market clearance. Considering that only a mere one-third of adults in the United States [197] have reported visiting the eyecare clinic at least once a year, as recommended by the American Optometric Association [198], the commercialization of at-home retinal imaging systems would mark a significant advancement in the prevention of glaucoma.

Lastly, one of the most fundamental challenges that remains is the difficulty of visualizing transparent and sparsely distributed RGCs in a label-free manner [199]. While fluorescence imaging has enabled the precise identification of individual cells in animal models, translating these techniques to human patients remains challenging due to safety, regulatory, and practicality concerns. As such, the development of high-resolution, label-free approaches, such as phase-sensitive OCT, intrinsic signal imaging, and advanced adaptive optics [200], can be more beneficial for clinical applications. Achieving reliable detection and quantification of RGCs without exogenous agents will represent a major milestone toward routine screening and longitudinal tracking of neurodegenerative retinal diseases.

## Data Availability

Not applicable.
